# Automated Pretreatment Thoracic CT-Based Body Composition Analysis Predicts Progression-Free Survival in Head and Neck Cancer

**DOI:** 10.3390/jcm15114169

**Published:** 2026-05-28

**Authors:** Frederic Jungbauer, Clara Arndt, Lena Huber, Anne Lammert, Nicole Rotter, Claudia Scherl, Elena Seiz, Farroch Vahidi Noghani, Stefan O. Schoenberg, Johannes Haubold, Sonja Ludwig, Annette Affolter, Fabian Tollens, Dominik Nörenberg, Johannes M. Ludwig

**Affiliations:** 1 Department of Otorhinolaryngology, Head and Neck Surgery, University Medical Center Mannheim, Medical Faculty Mannheim of Heidelberg University, 68167 Mannheim, Germany; frederic.jungbauer@umm.de (F.J.);; 2 Department of Otolaryngology, Head and Neck Surgery, Campus Klinikum Bielefeld Mitte, University Hospital OWL of Bielefeld University, 33604 Bielefeld, Germany; 3 Department of Radiology and Nuclear Medicine, University Medical Center Mannheim, Medical Faculty Mannheim of Heidelberg University, 68167 Mannheim, Germany; 4 Department of Diagnostic and Interventional Radiology and Neuroradiology, University Hospital Essen, University of Duisburg-Essen, 45147 Essen, Germany

**Keywords:** head and neck cancer (HNC), body composition analysis (BCA), sarcopenia, subcutaneous adipose tissue (SAT), computed tomography (CT) imaging

## Abstract

**Background/Objectives**: To evaluate the prognostic significance of automated, volumetric body composition analysis (BCA) derived from pretreatment thoracic computed tomography (CT) scans in patients with head and neck cancer (HNC). **Methods**: We retrospectively assessed 160 patients (median age: 63 years; 26.9% women) undergoing primary treatment. BCA quantified various tissue volumes, including bone (B), skeletal muscle (SM), and subcutaneous adipose tissue (SAT). Optimal sex-specific cutoffs for BCA metrics were established via maximally selected log-rank tests. Internal validation of BCA cutoffs was conducted via bootstrap resampling. Kaplan–Meier survival analysis and Cox proportional hazards modeling were used to investigate progression-free survival (PFS). **Results**: The median PFS for all patients was 51.7 months (95% confidence interval (CI): 31.4–68.8). Among the continuous BCA parameters, only SM/B was significant across the total cohort (hazard ratio (HR): 0.23; 95%CI: 0.12–0.46; *p* < 0.0001, males (*p* = 0.0009), females (*p* = 0.004)). Internal validation of gender-specific cutoffs demonstrated strong-to-intermediate stability for SM/B across both sexes and for SAT/B in males. In contrast, SAT/B exhibited only weak stability among female participants. In univariate PFS analysis, dichotomized SM/B, SAT/B, Union for International Cancer Control (UICC) stage, Eastern Cooperative Oncology Group (ECOG) status, higher body mass index (BMI), normal albumin, and Charlson Comorbidity Index were identified as significant predictors of PFS. Multivariable analysis identified high SM/B (HR: 0.53; 95% CI: 0.3–0.93; *p* = 0.026) and high SAT/B (HR: 0.58; 95% CI: 0.35–0.95; *p* = 0.029) as independent prognostic factors, alongside lower UICC stage (*p* = 0.045) and lower Charlson Comorbidity Index (*p* = 0.038). Patients with high SM/B and SAT/B ratios had the longest median PFS (65.9 months, 95%CI: 51.7–.), compared to 36.4 months (95%CI: 19.4–.) for high SM/B or SAT/B and 12.6 months (95%CI: 4.2–25.1) for low SM/B and SAT/B (*p* < 0.0001). **Conclusions**: Although the BCA parameters SM/B and, to a lesser extent, SAT/B appear to be promising biomarkers, external validation and investigation within well-defined patient subgroups are warranted to establish their generalizability in clinical practice.

## 1. Introduction

As the seventh most common cancer type worldwide, the incidence of head and neck cancer (HNC) is characterized by a significant and ongoing upward trend. Research by Deng et al. highlights a divergent trend: while regional mortality rates are declining, the total diagnostic volume remains high. This surge, driven by shifting demographics and specific subtypes, is projected to increase the global burden through 2030 [1]. Conventional first-line treatment strategies for these diseases generally involve combining multiple modalities, including surgical intervention, radiation therapy, and systemic drug therapy [2]. However, accurately predicting treatment success remains clinically challenging.

The significance of body composition is gaining recognition in cancer patients, particularly the role of sarcopenia, a condition characterized by progressive loss of skeletal muscle mass and strength. Sarcopenia has emerged as a critical prognostic factor in oncology, affecting treatment tolerance, quality of life, and survival rates across various cancer types, including HNC [3,4,5]. Patients diagnosed with HNC exhibit a heightened propensity to develop sarcopenia. This elevated risk can be attributed to heightened vulnerability to malnutrition and the presence of related health conditions [6]. Consequently, elevated sarcopenia rates ranging from 6.6% to 77.14% are often observed in these patients, depending on the population and assessment method [7,8,9].

Historically, radiological body composition assessments have necessitated the manual segmentation of computed tomography (CT) scans at specific anatomical landmarks, such as the third lumbar or cervical vertebrae, or, less frequently, the first or second thoracic vertebrae. This manual approach is often too labor-intensive and inefficient for standard clinical workflows [3,7,10,11]. However, the emergence of automated tools, particularly those powered by convolutional neural networks, has revolutionized this field. These advancements have facilitated high-speed, consistent, and uniform quantification of muscle, adipose, and bone tissues throughout the entire CT volume [12,13]. Previous studies have demonstrated the prognostic relevance of whole CT-scan muscle volume in various cancers, including colorectal cancer [14] and recurrent and/or metastatic HNC undergoing immunotherapy [15].

Although research on body composition in HNC frequently utilizes abdominal imaging, thoracic and cervical scans are more commonly employed in routine clinical practice. Given the propensity of cervical measurements to be influenced by factors such as local tumor invasion of fat and muscle, dental artifacts, image degradation, and treatment-induced changes that affect follow-up analysis, thoracic imaging is likely a more reliable modality for consistent assessment. However, the prognostic significance of pretreatment body composition parameters derived from whole-thoracic CT scans in the first-line setting remains to be evaluated.

The objective of this study was to assess the prognostic value of automated volumetric body composition analysis from pretreatment thoracic CT scans for predicting progression-free survival (PFS) in patients newly diagnosed with HNC.

## 2. Materials and Methods

Between June 2017 and June 2023, 314 patients received primary treatment for HNC at our institution. The final analysis was conducted on 160 patients for whom baseline thoracic CT scans were accessible. Patient clinical and laboratory parameters were extracted from electronic medical records, with 20 February 2026, as the cutoff date for censoring follow-up data. The Institutional Review Board (IRB) granted approval for this retrospective investigation (IRB #: 2024-892), with a waiver of informed consent provided.

### 2.1. Automated Body Composition Analysis

**Figure 1:**
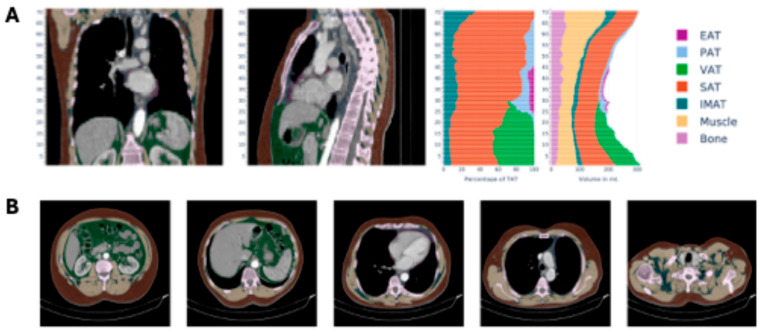
Exemplary visualization of a fully automated body composition segmentation performed on a thoracic computed tomography (CT) scan. (**A**) Coronal and sagittal CT views depict color-coded tissue distributions alongside corresponding tissue distribution maps. (**B**) Representative axial slices illustrating segmented tissues. Abbreviations: EAT, epicardial adipose tissue; IMAT, intra- and intermuscular adipose tissue; PAT, pericardial adipose tissue; SAT, subcutaneous adipose tissue; TAT, total adipose tissue; VAT, visceral adipose tissue.

Body composition was assessed employing a fully automated algorithm for CT image interpretation, as previously described in detail [13,16,17]. The corresponding open-source implementation is available at https://github.com/UMEssen/Body-and-Organ-Analysis (version 0.1.3; accessed on 9 December 2024). This method employs a pretrained convolutional neural network to quantify 3D volumes of different body tissue compartments based on standard thoracic CT scans. As illustrated in Figure 1, the algorithm determines the volume of various body components. Volumetric data is captured for skeletal muscle (SM) and bone (B), in addition to a detailed breakdown of adipose tissue, including the subcutaneous (SAT), visceral (VAT), pericardial (PAT), epicardial (EAT), and intra- and intermuscular (IMAT) compartments. To compensate for individual differences in patient anatomy and scan extent, the algorithm automatically segments the thoracic cavity, extending partially into the upper abdomen. For internal normalization, body composition volumes were standardized relative to the bone volume.

### 2.2. Statistics

Median progression-free survival (PFS) and median overall survival (OS) were calculated using the Kaplan–Meier method, and intergroup differences were evaluated by applying the log-rank test. Normality of body composition parameters was evaluated independently for each sex using the Shapiro–Wilk test; *p*-values < 0.05 indicated non-normality. Sex-specific comparisons were conducted using Mann–Whitney U tests because the data for each parameter deviated from normality in at least one sex group.

We utilized a maximally selected log-rank statistic to determine the optimal cut-off points for dichotomizing body composition metrics. Optimal thresholds were identified by testing percentiles from the 20th to the 80th percentile and selecting the cutoff point that yielded the minimum two-sided log-rank *p*-value. Sex-stratified cutoffs were applied to parameters that showed significant sex differences. Internal validation was performed using bootstrapping with 1000 resamples to evaluate the stability of the selected cutoffs and to estimate the apparent optimism in model performance. Stable cutoffs were defined by close agreement between the original cutoff and bootstrap median, narrow bootstrap percentile intervals, and higher bootstrap stability.

Continuous variables were categorized using established clinical thresholds: C-reactive protein (CRP) and albumin were dichotomized at the lower limit of their normal ranges. Body mass index (BMI, kg/m^2^) was classified based on both the lower and upper boundaries of the normal range (≥18.5–24.9 kg/m^2^).

Prognostic indicators were identified using univariate and multivariate Cox proportional hazards regression models to estimate hazard ratios (HRs) and 95% confidence intervals (CIs). To be included in the multivariate analysis, variables had to meet two criteria. First, they had to be statistically significant in the univariate analysis. Second, they had to demonstrate low overall multicollinearity. To assess multicollinearity, we conducted a variance inflation factor (VIF) analysis and represented categorical data using dummy coding. To interpret the degree of multicollinearity, the following VIF cut-offs were applied: low (<5), moderate (5–10), and high (≥10). The next step involved systematically eliminating parameters with VIF values ≥ 10. This process was carried out methodically until all parameters had VIF values < 5. Missing values for covariates in the multivariate analysis were addressed by treating missingness as a distinct category within the model. Pearson’s correlation coefficients were used to examine associations between variables. Given the study’s exploratory design, no adjustments for multiple comparisons (alpha error correction) were made, and significance was established at *p* < 0.05. Data analysis was performed using JMP 19.05 (SAS Institute Inc., Cary, NC, USA) or R v4.4.3. AI-assisted language editing was used to improve grammar, clarity, and readability; all scientific content, analyses, and final wording were reviewed and approved by the authors.

## 3. Results

### 3.1. Baseline Characteristics

Among the 160 patients studied (median age 63; 26.9% female), the oropharynx was the most common primary tumor site (32.5%). Other frequent locations included the larynx and oral cavity, accounting for 14.4% and 13.8% of the group, respectively. Almost one quarter of the patients (23.8%) presented with early-stage disease (Union for International Cancer Control (UICC) Stage 0–I). Table 1 summarizes the patients’ baseline characteristics.

### 3.2. Progression-Free Survival Analysis

Disease progression or mortality occurred in 72 individuals, with a median time of 9.6 months (range: 0.36–69.8 months) recorded from the initiation of treatment. The remaining patients were lost to follow-up or censored from subsequent observation after a median time of 35.8 months (range: 2.4–82.4 months). The median PFS of all patients was 51.7 months (95% CI: 31.4–68.8). PFS rates at 6 months, 1, 2, 3, 4, and 5 years were 84.2% (95% CI: 77.6–89.1), 73.8% (95% CI: 66.1–80.0), 62.5% (95% CI: 54.2–69.7), 58.2% (95% CI: 49.7–65.8), 52.2% (95% CI: 42.6–61.0), and 40.9% (95% CI: 27.3–54.1), respectively.

### 3.3. Prognostic Impact of Body Composition Metrics on Progression-Free Survival

Except for EAT/B, all tested body composition parameters differed significantly between sexes (Supplementary Table S1). When applying a Cox proportional hazards model to continuous body composition metrics, only SM/B was identified as a significant independent predictor of PFS. Higher SM/B values were associated with significantly lower hazard ratios in the total cohort (HR: 0.234; 95% CI: 0.12–0.46; *p* < 0.0001), as well as in male (HR: 0.269; 95% CI: 0.124–0.585; *p* = 0.0009) and female patients (HR: 0.067; 95% CI: 0.01–0.43; *p* = 0.0043). No other continuous body composition parameter demonstrated a statistically significant association with PFS (Table 2).

Following dichotomization of body composition parameters, only patients with SM/B (*p* = 0.0001) and SAT/B (*p* = 0.0087) values above the defined cutoffs had a significantly lower hazard ratio and longer PFS (Table 3 and Supplementary Table S2). When stratified by sex, SM/B maintained its prognostic significance for both groups, with higher muscle mass correlating to substantially longer median PFS for males with 65.9 months (95% CI: 51.7–.) vs. 22.8 months (95% CI: 12.7–46.3; *p* = 0.0003) and females with 54.2 months (95% CI: 38.9–54.2) vs. 6.7 months (95% CI: 1.4–.; *p* = 0.0009).

A higher SAT/B ratio was strongly associated with improved PFS in males with 65.9 months (95% CI: 36.5–.) vs. 12.6 months (95% CI: 2.3–25.1; *p* < 0.0001) but was not seen in females (38.9 months (95% CI: 1.4–.) vs. 54.2 months (95% CI: 14.6–54.2)) (*p* = 0.39). Correlation analysis revealed only a weak association between SAT/B and SM/B across the entire cohort (r^2^ = 0.032; *p* = 0.024). This negligible correlation persisted upon sex stratification in female (r^2^ = 0.012; *p* = 0.023) and male patients (r^2^ = 0.019; *p* < 0.0001).

### 3.4. Internal Validation of SM/B and SAT/B Cutoffs by Sex

Bootstrap internal validation suggested that the SM/B cutoff was reasonably stable in both male and female patients, with close agreement between the maximally selected log rank test and the bootstrap-derived cutoffs: In males, the SM/B cutoff of 1.9888 was nearly identical to the bootstrap median cutoff of 1.987, with a bootstrap stability of 41.3% and a 95% bootstrap CI of 1.719–2.130. In females, the internal stability of SM/B was higher, with a cutoff of 1.4671, a bootstrap median of 1.482, a bootstrap stability of 62.6%, and a narrower 95% bootstrap CI of 1.452–1.738.

In contrast, SAT/B showed only moderate stability in males and poor stability in females: Among male patients, the SAT/B cutoff of 0.8862 shifted to a bootstrap median of 1.081, with a bootstrap stability of 44.7% and a 95% bootstrap CI of 0.845–1.789, indicating moderate reproducibility. Among female patients, the SAT/B cutoff of 2.9037, a bootstrap median of 2.287, a low bootstrap stability of 31.0%, and a wide 95% bootstrap CI of 1.366–3.456 indicated limited stability.

### 3.5. Uni- and Multivariate Analysis of Prognostic Risk Factors for PFS

In addition to the two body composition parameters, prolonged PFS was observed in patients with a lower UICC stage, higher BMI, lower Charlson Comorbidity Index, and normal albumin levels (Table 3). In the VIF analysis of significant factors, BMI showed high redundancy (VIF 13.98). After its exclusion, all remaining VIF values were <5 (Supplementary Table S4). In the subsequent multivariate analysis, higher SM/B (HR: 0.53; 95% CI: 0.3–0.93; *p* = 0.026), higher SAT/B (HR: 0.58; 0.35–0.95; *p* = 0.029), lower UICC stage (*p* = 0.045), and lower Charlson Comorbidity Index (*p* = 0.038) were identified as independent prognostic factors (Table 3).

### 3.6. Comparison of BMI with SM/B and SAT/B

When comparing stratified BMI to dichotomized SM/B in a Cox proportional hazards analysis, the overall effect size was greater for SM/B (3.4, *p* = 0.0006) than for BMI (1.5, *p* = 0.03). Furthermore, in a direct comparison of continuous variables, only SM/B remained an independent predictor of PFS (HR: 0.21; 95% CI: 0.09–0.47; *p* = 0.0002), whereas BMI showed no significant independent association (HR: 0.99; 95% CI: 0.93–1.05; *p* = 0.73). Moreover, a high SM/B ratio was significantly associated with a reduced HR and prolonged PFS across normal and high BMI: In the normal-BMI group, an elevated SM/B ratio yielded an HR of 0.39 (95% CI: 0.18–0.85; *p* = 0.018), with a median PFS of 54.2 months vs. 15.9 months for the low-ratio group (*p* = 0.014). Similarly, in high-BMI patients, a high SM/B ratio was associated with a significantly lower risk of progression (HR: 0.35; 95% CI: 0.16–0.77; *p* = 0.009) and longer PFS, with the median not reached vs. 36.4 months in the low-ratio cohort (*p* = 0.007). In contrast, no superiority of SAT/B over BMI was observed.

### 3.7. Prognostic PFS Substratification of SM/B and SAT/B Across Clinical Parameters

Subgroup analyses revealed that the SM/B ratio provided further risk stratification across all ECOG performance status categories. Patients with higher SM/B ratios had significantly longer PFS than those with lower ratios. This association was consistent for patients with ECOG 0 (median not reached vs. 22.4 months; 95% CI: 9.6–57.3; *p* < 0.0001), ECOG 1 (22.1 months; 95%CI: 9.1–46.3 vs. 54 months; 95% CI: 22.6–65.9; *p* = 0.037), and ECOG ≥ 2 (3.8 months; 95% CI: 1.34–. vs. 0.36 months; 95% CI: .–.; *p* = 0.0005). For the SAT/B ratio, prognostic value was restricted to patients with ECOG 1 status, in which higher ratios predicted a significantly longer median PFS (51.7 months; 95% CI: 30.8–. vs. 15.9 months; 95% CI: 4.6–54.2; *p* = 0.01).

When stratified by UICC stage, the additional prognostic utility of the SM/B and SAT/B ratios was limited. Significant substratification was observed for the SM/B ratio only in UICC stage III, with a median not reached; 95% CI: 28.7–. vs. 12.2 months; 95% CI: 3.7–.; *p* = 0.039. Conversely, the SAT/B ratio reached significance only in stage IV, with a median PFS of 30.8 months; 95% CI: 13.0–. compared to 12.6 months; 95% CI: 6.7–25.1; *p* = 0.036.

For the Charlson Comorbidity Index, a significant substratification was observed for SM/B across all groups: 1–2 (median not reached; 95% CI: 8-. vs. 5.6 months; 95% CI: 1.7–9.6; *p* = 0.006), 3–4 (65.9 months; 95% CI: .-. vs. 22.1 months; 95% CI: 12.6-.; *p* = 0.004), and ≥5 (51.7 months; 95% CI: 36.5-.) vs. 19.4 months; 95% CI: 7.5–36.4; *p* = 0.026).

Regarding pretreatment serum albumin levels, a significant stratification benefit was observed exclusively in the normal albumin subgroup. For the SM/B ratio, the median PFS was not reached (95% CI: 54.2–.) vs. 19.6 months (95% CI: 12.6–57.3; *p* < 0.0001) for high and low values, respectively. For the SAT/B ratio, the median PFS was not reached (95% CI: .-.) in the high-ratio cohort, compared with 54.2 months in the low-ratio cohort (95% CI: 9.6–68.9; *p* = 0.02).

### 3.8. Prognostic Impact of SM/B and SAT/B Across Treatment Modalities and Therapeutic Intent

Dichotomized SM/B demonstrated the most pronounced effect in patients who underwent surgery alone (HR: 0.13; 95% CI: 0.04–0.40; *p* = 0.0003), followed by those who received primary radio(chemo)therapy (HR: 0.39; 95% CI: 0.18–0.86; *p* = 0.02). In contrast, the treatment effect failed to reach statistical significance among patients who underwent surgical intervention followed by adjuvant radio(chemo)therapy (HR: 0.57; 95% CI: 0.23–1.37; *p* = 0.21). For dichotomized SAT/B, statistical significance was reached only in the radio(chemo)therapy group (HR: 0.38; 95% CI: 0.19–0.75; *p* = 0.006).

SM/B and SAT/B reached significance exclusively in the curative intent group (Supplementary Table S3). For palliative intent (n = 13), the results remained non-significant (*p* > 0.41) across both continuous and dichotomized analyses.

### 3.9. Combined Body Composition Analysis Further Stratifies PFS

**Figure 2:**
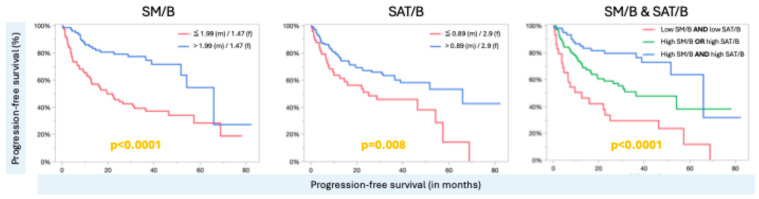
Kaplan–Meier estimates of progression-free survival stratified for SM/B, SAT/B, and SM/B + SAT/B. Significant differences were observed across all groups in the right panel (*p* ≤ 0.012).

To explore the potential for enhanced PFS risk stratification by body composition parameters, a simple scoring system based on SM/B and SAT/B was assessed. Patients exceeding the cutoffs for both SM/B and SAT/B demonstrated the best outcomes, with a median PFS of 65.9 months (95% CI: 51.7–.). Conversely, patients exhibiting a diminished SM/B or SAT/B ratio had a median PFS of 36.4 months (95% CI: 19.4–.). Patients with low values for both markers demonstrated the shortest PFS, at a median of 12.6 months (95% CI: 4.2–25.1; *p* < 0.0001) (Figure 2). Subgroup analyses confirmed that these significant differences were consistent across all comparisons (*p* ≤ 0.012). In sex-stratified analyses, the scoring system effectively differentiated risk among men (*p* < 0.0001), whereas no significant differences in PFS were observed within the female subgroup (*p* = 0.37)

### 3.10. Overall Survival Analysis

Median OS was not reached in the entire population, with a median follow-up of 32.2 months. Consistent with the PFS findings, an SM/B value above the sex-specific cutoff was associated with significantly superior OS (log-rank *p* = 0.0002). In the high SM/B group, 1- and 3-year survival rates were 93.6% (95% CI: 85.3–97.3%) and 90.6% (95% CI: 81.2–95.4%), respectively. Conversely, survival rates for patients below the cutoff were markedly lower, at 77.0% (95% CI: 64.4–83.7%) at 1 year and 62.0% (95% CI: 49.2–72.5%) at 3 years. Similar trends were observed for SAT/B. In the high-value group, 1- and 3-year survival rates were 89.8% (95% CI: 82.3–94.2) and 82.5% (95% CI: 73.1–88.9), respectively. By comparison, patients with low SAT/B values had survival rates of 75.0% (95% CI: 60.2–85.0) and 62.0% (95% CI: 45.8–74.6) (log-rank *p* = 0.0006).

## 4. Discussion

This study highlights the overall prognostic potential of automated body composition profiling for general risk stratification in patients with HNC receiving first-line therapy. While direct comparisons with existing literature are limited by population heterogeneity and methodological differences, the broad prognostic utility of parameters derived from skeletal muscle and subcutaneous adipose tissue is well supported.

A large-scale meta-analysis of 18 studies identified sarcopenia as a significant risk factor for disease-free survival in patients treated with surgery (HR 2.59; 95% CI: 1.56–4.31) and in those undergoing radiotherapy only (HR 1.56; 95% CI: 1.24–1.97) [11]. Despite differences in study endpoints, partly due to the inclusion of palliative cases, our results are consistent with this meta-analysis, demonstrating low skeletal muscle mass as a strong predictor of PFS across the entire cohort (HR: 2.89; 95% CI: 1.74–4.79). This effect was also observed in the surgical cohort (HR: 7.6; 95% CI: 2.5–23.3), suggesting that muscle mass affects outcomes in this subgroup. Notably, this association did not reach statistical significance within the radiotherapy-only group (HR: 1.3; 95% CI: 0.27–6.7). This discrepancy is likely attributable to the modest sample size (n = 14) in the latter subgroup, which may have rendered the analysis underpowered. Several studies have reported significantly higher PFS hazard ratios for sarcopenic patients undergoing radiotherapy ± chemotherapy. These ranged from 1.65 (*p* = 0.03) when measured at the level of the third cervical vertebra [18] and 2.26 (95% CI: 1.39–3.66; *p* = 0.001) at the level of the third lumbar vertebra [19] to 2.79 (95% CI: 1.52–5.13; *p* = 0.001) when measuring temporal muscle thickness [20]. These findings are generally consistent with this study’s results, with an HR of 2.6 (95% CI: 1.16–5.6) for patients with low SM/B values.

Previously, OS cutoffs in patients with recurrent and metastatic HNC treated with immunotherapy have been reported for SM/B (≤1.79 (m)/1.56 (f)) [15]. When the previously established thresholds were applied to this cohort, a significant survival benefit was observed in the higher SM/B group (log-rank test; *p* = 0.0004). Specifically, this group achieved a median PFS of 65.9 months (95% CI: 28.9–.), whereas those with lower SM/B values had a median of only 22.07 months (95% CI: 10.7–54.2). The cutoffs were also statistically significant in males and females, respectively. Furthermore, patients with SM/B values between the previously reported cutoffs and those established in this study showed no significant differences in outcomes compared with patients with low SM/B. Although the SM/B cutoffs demonstrated comparable prognostic value across the two distinct patient cohorts, further research is warranted to validate or refine these thresholds to ensure robustness. It should be noted that SAT/B was not identified as a prognostic factor in the previous study.

A recent meta-analysis identified higher subcutaneous adipose tissue, measured at the third lumbar level, as a favorable prognostic factor associated with a lowered risk of progression in patients receiving immunotherapy (HR: 0.82; 95% CI: 0.68–1.00; *p* = 0.049) [21]. Similarly, a large retrospective study of 881 HNC patients treated with radiotherapy demonstrated that a higher pretreatment SAT index at the third thoracic level was significantly associated with improved distant metastasis-free survival (HR: 0.65; *p* = 0.015) and locoregional control (HR: 0.758; *p* = 0.047) [22]. Notably, while that study [22] did not find skeletal muscle to be a significant prognostic indicator, our analysis identified it as a key factor in risk stratification.

To further explore the clinical utility of SM/B and SAT/B ratios, subgroup analyses were conducted by ECOG performance status, UICC stage, and the Charlson Comorbidity Index. Overall, SM/B demonstrated greater prognostic performance than SAT/B. While its highest utility was observed within ECOG performance status subgroups, it showed limited prognostic value across UICC stages.

Regarding BMI, our findings align with a recent systematic review and meta-analysis of the BMI paradox in HNC, which demonstrated that patients with a higher BMI achieve significantly better outcomes than those with a lower BMI [23]. However, it should be noted that BMI cannot differentiate among tissue types, each of which may have varying prognostic significance, as outlined in this study, which identified the independent prognostic value of SM/B and SAT/B. A direct comparison between SM/B and BMI indicated that SM/B was superior. This was also evidenced by its ability to significantly stratify PFS outcomes in patients, regardless of whether they had a normal or elevated BMI. This finding is supported by previous studies, which demonstrated that CT-based sarcopenia measurements were independently superior to BMI for PFS [18] and OS [24]. Thus, given that serum albumin was not statistically significant in the multivariate analysis, the SM/B ratio may be a comparable, or even more precise, prognostic marker than conventional nutritional assessments, such as BMI or serum albumin levels. However, more research is needed to confirm these assumptions.

Internal validation of body composition markers revealed moderate and high stability of SM/B in male and female patients, respectively. Furthermore, the narrow confidence intervals and high concordance between determined and bootstrap-derived cutoffs support SM/B as a potentially useful candidate for PFS risk stratification. Conversely, the high variability of SAT/B in females underscores a lack of prognostic reliability, rendering its clinical relevance negligible in this cohort. This likely explains the absence of significant stratification by SAT/B in women. Notably, this sex-specific distinction has not been previously documented in single-slice CT-based body composition analyses. Given the moderate stability of SAT/B observed in men, it is possible that anatomical differences, such as breast volume, contribute to this instability. However, further research is warranted to understand the reasons for the inferior performance in women. Overall, the data above establish SM/B as a preferable candidate for clinical risk stratification, offering greater consistency than SAT/B for patients undergoing volumetric chest CT assessment.

Internal normalization by bone volume was performed, as in previous studies on HNC [15], colorectal cancer [14], and pancreatic cancer [25]. Internal normalization by bone volume offers a distinct methodological advantage, relying exclusively on CT-derived metrics and thereby obviating the need for external anthropometric data. The validity of this technique is underpinned by the robust physiological correlation between muscle and bone volumes, enabling reliable evaluation of muscle mass variations [26,27]. Notably, this muscle-to-bone correlation is superior to that of muscle and traditional metrics, such as BMI and body height, which have been conventionally employed for normalization in previous studies [27].

In the present study, we used thoracic CT scans as they were more often available than abdominal scans. However, recent updates to the German guidelines for oropharyngeal and hypopharyngeal carcinoma now include baseline abdominal CT staging. This transition is expected to broaden access to abdominal data for subsequent body composition analysis [28]. Regarding abdominal imaging, it remains to be determined whether prognostic BCA factors identified on thoracic scans retain significance when measured abdominally and whether SAT/B has prognostic utility in women.

Evidence strongly supports implementing targeted nutritional interventions to optimize patient prognoses [29]. One study found that combining structured nutritional counseling with oral nutritional supplements significantly improves three-year overall survival rates compared to supplement administration alone (93.4% vs. 85.4%, *p* = 0.031) [30]. Additionally, systematic meta-analytic evidence regarding multimodal prehabilitation protocols underscores their clinical utility, demonstrating a 38% reduction in overall mortality [31]. Furthermore, exercise interventions significantly enhance functional capacity and quality of life; however, whether these improvements translate into a survival advantage remains to be established [32,33]. Mascarella et al. demonstrated that patients who recovered from CT-based measured sarcopenia, assessed at the third cervical vertebra, had survival rates comparable to those who never developed the condition. Conversely, patients with persistent or incident sarcopenia experienced significantly worse clinical outcomes [34]. Notably, as the study by Mascarella et al. did not implement any active nutritional or physical interventions, it remains to be determined whether such strategies can favorably modulate adverse CT-based body composition phenotypes and if this ultimately translates into improved patient outcomes.

Limitations: While this study and the broader literature underscore the overall prognostic significance of skeletal muscle and subcutaneous adipose tissue, our findings should be interpreted with caution due to several reasons limiting validity, mainly driven by a retrospective, single-center design, modest sample size, low frequency of clinical events, and a heterogeneous cohort encompassing diverse tumor sites, stages, and treatment regimens. Moreover, a potential selection bias must be acknowledged, as baseline CT scans were unavailable for all patients.

## 5. Conclusions

This study demonstrates that automated body composition analysis of baseline thoracic CT scans provides significant prognostic insights into HNC, identifying the SM/B and SAT/B ratios, alongside UICC stage and Charlson Comorbidity Index, as independent prognostic factors for PFS. Among the evaluated body composition markers, the SM/B ratio emerged as the most promising prognostic biomarker. However, external validation of findings in well-defined patient (sub)groups is required for further evaluation and to define and establish the clinical applicability of SM/B and SAT/B as prognostic markers.

## Figures and Tables

**Table 1:** Baseline demographic and clinical characteristics of the study population.

Baseline Characteristics	Patients
Sex	
Male	117 (73.1%)
Female	43 (26.9%)
Location of primary tumor	
Oropharynx	52 (32.5%)
Larynx	23 (14.4%)
Oral cavity	22 (13.8%)
Multi-level	17 (10.6%)
Nose	13 (8.1%)
Cancer of unknown primary (CUP)	8 (5.0%)
Parotid gland	7 (4.4%)
Nasopharynx	7 (4.4%)
Hypopharynx	7 (4.4%)
Sinuses	3 (1.9%)
Submandibular gland	1 (0.6%)
Tumor stage (UICC)	
0	6 (3.8%)
I	32 (20.0%)
II	23 (14.4%)
III	29 (18.1%)
IV	70 (43.8%)
p16-Status	
Positive	47 (29.4%)
History of substance use	
Tobacco	59 (36.9%)
Alcohol and Tobacco	53 (33.1%)
None	40 (25.0%)
Alcohol	8 (5.0%)
Therapy	
Primary RCT	47 (29.4%)
Surgery	41 (25.6%)
Surgery + RCT	33 (20.6%)
Surgery + RT	23 (14.4%)
Primary RT	14 (8.8%)
Primary CT	1 (0.6%)
Primary RT + Immunotherapy	1 (0.6%)
ECOG	
0	72 (45.0%)
1	73 (45.6%)
2	8 (5.0%)
3	2 (1.3%)
4	3 (1.9%)
Unknown	2 (1.3%)

Baseline patient characteristics and clinical data. Abbreviations: CT, chemotherapy; ECOG, Eastern Cooperative Oncology Group performance status; RCT, radiochemotherapy; RT, radiotherapy; UICC, Union for International Cancer Control.

**Table 2:** Association of continuous body composition parameters with progression-free survival.

Body Composition Parameter	All Patients		Male Patients		Female Patients	
	HR (95% CI)	*p*-Value	HR (95% CI)	*p*-Value	HR (95% CI)	*p*-Value
SM/B	0.234 (0.12–0.46)	<0.0001	0.269 (0.124–0.585)	0.0009	0.067 (0.01–0.43)	0.0043
TAT/B	0.913 (0.76–1.1)	0.33	0.786 (0.598–1.035)	0.09	1.073 (0.828–1.39)	0.59
IMAT/B	1.23 (0.41–3.72)	0.72	0.792 (0.191–3.288)	0.75	3.068 (0.386–24.4)	0.29
SAT/B	0.88 (0.69–1.12)	0.28	0.674 (0.444–1.025)	0.065	1.049 (0.763–1.4)	0.77
VAT/B	0.53 (0.14–2.1)	0.36	0.318 (0.066–1.532)	0.15	18.575 (0.372–927)	0.14
PAT/B	0.29 (0.007–12.41)	0.52	0.067 (0.001–5.77)	0.23	68.194 (0.03–155,693)	0.29
EAT/B	0.106 (0.0–4453)	0.68	<0.001 (0.0–43.49)	0.16	1,018,547.78 (0.001–.)	0.19

Univariate Cox proportional hazards analysis of body composition parameters for progression-free survival (PFS), stratified by the total population and sex. Abbreviations: B; Bone, EAT; Epicardial Adipose Tissue, HR; Hazard Ratio, IMAT; Intramuscular Tissue, PAT; Pericardial Adipose Tissue, SAT; Subcutaneous Adipose Tissue, SM; Skeletal Muscle, TAT; Total Adipose Tissue, VAT; Visceral Adipose Tissue; 95% CI, 95% confidence interval.

**Table 3:** Uni- and multivariate progression-free survival analysis of pretreatment factors.

				Univariate Analysis		Multivariate Analysis	
Groups		*n*	Median PFS in Months (95% CI)	HR (95% CI)	*p*-Value	HR (95% CI)	*p*-Value
Gender	Female	43	54.1 (22.4–54.2)	1	0.86	-	-
	Male	117	51.7 (30.8–68.8)	1.05 (0.6–1.83)		-	
Age	>70 years	49	65.9 (36.5–.)	1	0.18	-	-
	≤70 years	111	36.4 (16.7–57.3)	0.71 (0.44–1.17)		-	
UICC	0–I	38	57.3 (39.9–65.9)	1	0.0001	1	0.045
	II	23	51.7 (9.1–.)	3.57 (1.38–9.2)		2.6 (0.99–67.0)	
	III	29	NR (16.8–.)	2.4 (0.94–6.28)		1.5 (0.57–4.2)	
	IV	70	19.6 (12.1–46.3)	4.69 (2.1–10.48)		2.7 (1.7–6.4)	
p16	Not positive	113	46.3 (22.4–65.9)	1	0.1	-	-
	positive	47	NR (31.4–68.8)	0.63 (0.35–1.12)		-	
ECOG	0	72	68.8 (57.3–.)	1	0.0026	1	0.23
	1	73	46.3 (22.59–65.93)	1.55 (0.93–2.58)		1.4 (0.79–2.4)	
	2–4	13	3.74 (1.3–.)	4.47 (2.1–9.7)		2.6 (1.08–6.0)	
BMI (kg/m^2^)	<18.5	6	9.6 (4.1–.)	1	0.0005	-	-
	≥18.5–24.9	66	25.1 (13.0–46.3)	0.62 (0.22–1.74)		-	
	>24.9	85	NR (51.7–.)	0.25 (0.09–0.73)		-	
CCI	1–2	11	NR (8–.)	1	0.016	1	0.038
	3–4	67	65.93 (65.9–.)	1.35 (0.46–3.9)		2.3 (0.7–7.1)	
	≥5	81	36.4 (16.4–51.7)	1.5 (0.55–4.3)		1.4 (0.44–4.3)	
Albumin	≤3.4 g/dL	42	16.69 (6.7–46.3)	1	0.0029	1	0.64
	>3.4 g/dL	110	57.3 (36.5–.)	0.46 (0.28–0.75)		0.77 (0.45–1.3)	
CRP	Normal	65	56.2 (36.4–57.3)	1	0.2	-	-
	Elevated	60	26.4 (12.7–.)	1.4 (0.83–2.35)		-	
SM/B	≤1.9888 (m)/1.4671 (f)	68 (m) 12 (f)	19.6 (12.1–36.4)	1	<0.0001	1	0.026
	>1.9888 (m)/1.4671 (f)	49 (m) 31 (f)	65.9 (51.7–.)	0.35 (0.21–0.57)		0.53 (0.3–0.93)	
SAT/B	≤0.8862 (m)/2.9037 (f)	21 (m) 28 (f)	25.1 (10.0–57.3)	1	0.0087	1	0.029
	>0.8862 (m)/2.9037 (f)	96 (m) 15 (f)	65.9 (36.5–.)	0.53 (0.33–0.85)		0.58 (0.35–0.95)	

Univariate and multivariate progression-free survival analyses of pretreatment factors. The number of events that occurred per group is listed in Supplementary Table S5. Abbreviations: B, bone; BMI, body mass index; CCI, Charlson Comorbidity Index; CI, confidence interval; CRP, C-reactive protein; ECOG, Eastern Cooperative Oncology Group performance status; HR, hazard ratio; n, number of patients per group; PFS, progression-free-survival; SAT, Subcutaneous adipose tissue; SM, skeletal muscle; UICC, Union for International Cancer Control.

## Data Availability

The raw data supporting the conclusions of this article will be made available by the authors on request.

## Supplementary Materials

The following supporting information can be downloaded at: https://www.mdpi.com/article/10.3390/jcm15114169/s1, Table S1: Median values of body composition parameters stratified for sex; Table S2: Univariate Cox proportional hazard analysis of body composition parameters regarding progression-free survival; Table S3: Progression-free survival in patients with curative treatment intent stratified for SM/B and SAT/B; Table S4: Multicollinearity Assessment of PFS Predictors; Table S5: Number of PFS events occurred per subgroup; Table S6: Supporting minimal data.

## Abbreviations

The following abbreviations are used in this manuscript:

B	Bone
BCA	Body Composition Analysis
BMI	Body Mass Index
CI	Confidence Interval
CRP	C-reactive protein
CT	Computed Tomography
EAT	Epicardial adipose tissue
ECOG	Eastern Cooperative Oncology Group
HNC	Head and Neck Cancer
HR	Hazard Ratio
IMAT	Intra- and intermuscular adipose tissue
IRB	Institutional Review Board
OS	Overall survival
PAT	Pericardial adipose tissue
PFS	Progression-free survival
SAT	Subcutaneous adipose tissue
SAT/B	Subcutaneous adipose tissue-to-bone ratio
SM	Skeletal muscle
SM/B	Skeletal muscle-to-bone ratio
T1	First thoracic vertebra
T2	Second thoracic vertebra
T3	Third thoracic vertebra
TAT	Total adipose tissue
UICC	Union for International Cancer Control
VAT	Visceral adipose tissue
VIF	Variance Inflation Factor
