# Supplementary material for: Automated Pretreatment Thoracic CT-Based Body Composition Analysis Predicts Progression-Free Survival in Head and Neck Cancer
Source: J Clin Med. 2026 May 28;15(11):4169. doi: 10.3390/jcm15114169 (PMC13258539; doi:10.3390/jcm15114169)
Supplement: Supplementary file 1 [file jcm-15-04169-s001.zip › jcm-4275849-supplementary.pdf]

Supplementary tables for the Manuscript

Automated Pretreatment Thoracic CT-Based Body Composition Analysis Predicts Progression-Free Survival in Head and Neck Cancer

Supplementary Table S1. Median values of body composition parameters stratified for sex

**Body Composition Parameter**	**Median (IQR) all**	**Median (IQR) female**	**Median (IQR) male**	**p-value**
SM/B	1.882 (1.658–2.159)	1.702 (1.459–1.924)	1.932 (1.715–2.214)	**0.0001**
TAT/B	2.627 (1.913–3.446)	3.328 (2.619–4.336)	2.357 (1.836–3.086)	**<0.0001**
IMAT/B	0.51 (0.4–0.642)	0.607 (0.459–0.782)	0.469 (0.376–0.608)	**0.0009**
SAT/B	1.573 (1.097–2.187)	2.375 (1.734–3.061)	1.428 (1.022–1.802)	**<0.0001**
VAT/B	0.248 (0.158–0.368)	0.178 (0.108–0.283)	0.26 (0.171–0.408)	**0.0015**
PAT/B	0.126 (0.09–0.167)	0.11 (0.079–0.149)	0.132 (0.094–0.175)	**0.0227**
EAT/B	0.047 (0.036–0.068)	0.05 (0.038–0.073)	0.046 (0.035–0.065)	0.3263

The median values of standardized body composition ratios for the entire study cohort, male patients, and female patients with interquartile ranges (IQR) are presented. The Mann-Whitney U-test was employed to calculate differences between the sexes, given the non-normality of the distribution, at least for one sex in each group that was identified through the application of the Shapiro-Wilk test. Abbreviations: B (Bone), EAT (Epicardial Adipose Tissue), IMAT (Intramuscular Tissue), PAT (Pericardial Adipose Tissue), SAT (Subcutaneous Adipose Tissue), SM (Skeletal Muscle), TAT (Total Adipose Tissue), VAT (Visceral Adipose Tissue).

Supplementary Table S2. Univariate Cox proportional hazard analysis of body composition parameters regarding progression-free survival

Body Composition Parameters		Univariate Analysis	
		HR (95% CI)	p-value
SM/B	≤ 1.9888 (m) / 1.4671 (f)	1	**<0.0001**
	> 1.9888 (m) / 1.4671 (f)	0.35 (0.21–0.57)	
TAT/B	≤ 1.5826 (m) / 3.7501 (f)	1	0.1205
	> 1.5826 (m) / 3.7501 (f)	0.68 (0.41–1.11)	
IMAT/B	≤ 0.3276 (m) / 0.9552 (f)	1	0.641
	> 0.3276 (m) / 0.9552 (f)	0.89 (0.54–1.46)	
SAT/B	≤ 0.8862 (m) / 2.9037 (f)	1	**0.0087**
	> 0.8862 (m) / 2.9037 (f)	0.53 (0.33–0.85)	
VAT/B	≤ 0.15 (m) / 0.2974 (f)	1	0.2456
	> 0.15 (m) / 0.2974 (f)	0.75 (0.46–1.22)	
PAT/B	≤ 0.0926 (m) / 0.1388 (f)	1	0.5874
	> 0.0926 (m) / 0.1388 (f)	0.88 (0.54–1.42)	

Univariate Cox proportional hazard analysis of body composition parameters regarding progression-free survival. Patients were dichotomized using sex-specific cutoffs (male cutoff applied to males, female cutoff applied to females) and then analyzed together. Abbreviations: B (Bone), EAT (Epicardial Adipose Tissue), HR (Hazard Ratio), IMAT (Intramuscular Tissue), PAT (Pericardial Adipose Tissue), SAT (Subcutaneous Adipose Tissue), SM (Skeletal Muscle), TAT (Total Adipose Tissue), VAT (Visceral Adipose Tissue).

Supplementary Table S3: Progression-free survival in patients with curative treatment intent stratified for SM/B and SAT/B.

Marker	Patient Group	Median PFS (95% CI)	Log-rank p-value	HR (95% CI)	HR p-value
SM/B	> 1.9888 (m) / 1.4671 (f)	65.9 months (51.7–.)	<0.0001	0.22 (0.10–0.45)	<0.0001
	≤ 1.9888 (m) / 1.4671 (f)	22.8 months (12.6–46.3)			
SAT/B	> 0.8862 (m) / 2.9037 (f)	65.9 months (38.9–.)	0.005	0.49 (0.29–0.82)	0.006
	≤ 0.8862 (m) / 2.9037 (f)	28.7 months (10–57.3)			

Supplementary Table S3: Progression-free survival in patients with curative treatment intent stratified for SM/B and SAT/B.

Supplementary Table S4: Multicollinearity Assessment of PFS Predictors

**Variable**	**VIF (with BMI)**	**VIF (after BMI removal)**
BMI (<18.5 / normal / >24.9)	13.98	---
SAT/B (high vs. low)	5.77	2.87
Charlson CI (1-2 / 3-4 / 5+)	5.41	4.83
Albumin (≥34 vs. <34)	4.01	3.59
UICC (0-I / II / III / IV)	2.94	2.75
SM/B (high vs. low)	2.61	2.26
ECOG (0 / 1 / 2-4)	2.50	2.46

Supplementary Table S4: Variance inflation factor analysis demonstrating multicollinearity among progression-free survival predictors before and after removal of body mass index (BMI). VIF thresholds: <5 = low multicollinearity, 5-10 = moderate multicollinearity, ≥10 = high multicollinearity. Abbreviations: BMI, body mass index; CI, Charlson Comorbidity Index; ECOG, Eastern Cooperative Oncology Group performance status; HR, hazard ratio (95% confidence interval); SAT/B, subcutaneous adipose tissue to bone ratio; SM/B, skeletal muscle to bone ratio; UICC, Union for International Cancer Control stage.

Supplementary Table S5: Number of PFS events occurred per subgroup

Groups		Patients per Group	Number of PFS events occurred
Gender	Female	43	17
	Male	117	54
Age	>70 years	49	24
	≦70 years	111	47
UICC	0-I	38	7
	II	23	11
	III	29	11
	IV	70	42
p16	Not positive	113	56
	positive	47	15
ECOG	0	72	25
	1	73	36
	2-4	13	9
BMI (kg/m^2)	<18.5	6	4
	≥18.5–24.9	66	40
	>24.9	85	25
CCI	1-2	11	4
	3-4	67	23
	≥ 5	81	44
Albumin	≦3.4 g/dl	42	27
	>3.4g/dl	110	41
CRP	Normal	65	27
	Elevated	60	31
SM/B	≦1.9888 (m) / 1.4671 (f)	68 (m) 12 (f)	48
	>1.9888 (m) / 1.4671 (f)	49 (m) 31 (f)	22
SAT/B	≦0.8862 (m) / 2.9037 (f)	21 (m) 28 (f)	28
	>0.8862 (m) / 2.9037 (f)	96 (m) 15 (f)	43

Supplementary Table S5: Numbers of PFS events observed events per group of all factors from Table 3. Abbreviations: B, bone; BMI, body mass index; CCI, Charlson Comorbidity Index; CRP, C-reactive protein; ECOG, Eastern Cooperative Oncology Group performance status; PFS, Progression-free-survival; SAT, Subcutaneous adipose tissue; SM, Skeletal muscle; UICC, Union for International Cancer Control.
